# Adolescent gambling behavior: a gender oriented prevention strategy is required?

**DOI:** 10.1186/s13052-022-01309-3

**Published:** 2022-07-15

**Authors:** Alessandra Buja, Milena Sperotto, Bruno Genetti, Paolo Vian, Fabio Vittadello, Elisabetta Simeoni, Chiara Zampieri, Vincenzo Baldo

**Affiliations:** 1 Department of Cardiological, Thoracic, Vascular Sciences and Public Health, University of Padua, Padua, Italy; 2 Explora Center of Research and Statistical Analysis, Vigodarzere, PD Italy; 3 Technical Scientific and General Affairs Section, Department for Anti-Drug Policies, Prime Minister’s Office, Rome, Italy

**Keywords:** Gambling, Adolescents, Gender differences, Prevention, Risk factors

## Abstract

**Background:**

Studies published on gender-related differences in the gambling behavior of adolescents have focused mainly on psychological and social factors. The aim of this study was to develop separate risk factor models for male and female adolescents, considering the environmental, psychological, behavioral and socio-economic factors related to their gambling.

**Methods:**

A survey was conducted through a questionnaire developed on a dedicated web site in 2014 on a representative sample of the Italian 15–19-years-old population, including 34,922 students attending 438 secondary schools. The SOGS-RA questionnaire was used to measure gambling behavior. To verify the risk factors associated with gambling a logistic regression stratified by gender was performed.

**Results:**

In our representative sample of Italian adolescents nationwide, the prevalence of each level of gambling was higher in males than in females. The logistic regression stratified by gender found that for both genders, gambling was positively associated with internet surfing, playing sports, getting into a fight, having unprotected sex, pulling stunts, drinking alcohol at least once in the previous month, having not a satisfactory relationship with teachers, receiving pocket money from parents, spending each week much money and having someone in the family (father, sister/brother, other relatives) who gambles. On the other hand, having poor or average school marks, going to ED in the previous year, smoking at least once in the previous month, having dissatisfied with relationships with father and having a lower family income than their friends was only associated with gambling in boys. Having an accident or injury in the previous year and having a mother who gambled was associated only in girls with higher odd of at risk or problem gambling behavior. A low psychological distress is protective only in girls for risk of gambling.

**Conclusions:**

Understanding the gender-related differences, and how they emerge in younger people at the start of their gambling careers, can suggest how best to educate individuals, families and the community on the topic of gambling. Programs to prevent substance use and abuse should be multifaceted, and include efforts to prevent gambling with a gender perspective approach.

## Background

Gambling in adolescence is a growing public health problem. Traditional forms of gambling were generally considered an adult activity, but today’s youth are not immune to their appeal, and have grown up in a time that offers an abundance of gambling opportunities [1, 2]. It has been demonstrated that any gambling behavior, however minimal, is associated with other risk-taking behavior - such as substance use - and that adolescents’ “level of gambling risk” lies along a continuum, rather than falling into separate categories [3].

Gambling in adolescence is more common among males than females, and boys are more at risk of developing gambling problems than girls [4, 5]. Men with gambling problems also typically report having started to gamble in adolescence, whereas women tend to start later in life [6].

It has been demonstrated that what drives the differences between male and female gambling among adults is their motivation: women often reported gambling as an escape from their problems, to relieve stress and boredom; men were more likely to gamble for social reasons, for general entertainment, and to demonstrate their skills as players, or in an attempt to become wealthy from their win [7].

The very few studies published on gender-related differences in the gambling behavior of adolescents have focused mainly on psychological and social factors. A study performed on a sample of 12- to 18-year-olds found - in a not at risk gambling group - that depression was more likely to afflict female [8]. It has also been suggested that parents’ gambling behavior and family disharmony can have a role in problem gambling among adolescent females, whereas males are more likely to be influenced by their peer group [9]. A number of studies correlated adolescent problem gambling with poor school performance and school dropout, but gender-related differences were explored by only one study, which found that boys more frequently reported problems in their academic life [4, 8, 10]. Thus considerable amount of research has been conducted on gambling, also in adolescence, but relatively scarce and fragmented attention has been paid to the gender-related differences and similarities in the type of gambling, and the environmental, socio-economic and behavioral factors associated with gambling in this age group. Although risk factors cannot presume causation, identifying them enables high-risk groups to be recognized and targeted for prevention, early intervention and treatment strategies [11, 12]. A gendered understanding of these risk factors can inform the design of public health campaigns and the promotion of support services appropriately targeted to each gender.

The aim of this study on a large Italian sample of secondary-school students was to elucidate a comprehensive risk factor models taking into account environmental, psychological, behavioral, and socio-economic variables influencing their gambling behavior, to develop health promotion programs with a gender perspective approach.

## Methods

The sample population was drawn from the SPS-DAP (The Department for Anti-drug Policies’s Student Population Survey), a student population survey conducted in Italy during the first half of 2014 by the Department for Anti-drug Policies in collaboration with the Ministry of Education, Universities and Research, and with the participation of the Regional Representatives for Health Education. Full details of the design of the SPS-DAP have been published elsewhere (Presidenza del Consiglio Dei Ministri – Dipartimento Politiche Antidroga, 2013). For the purposes of the present study, the survey is briefly described below.

### Sample

The sample refers to the Italian student population between 15 and 19 years of age, sampled using a two-stage procedure that selected first a set of secondary schools, and then a set of students attending the schools concerned. The units (schools) selected in the first stage were stratified by region and type of school. The statistical units for the survey were represented by all the students attending each of the classes forming part of the sample, selected using a clustering method. The participation of schools and of the students in the study was optional: 70.8% of all selected schools participated in the survey (438 schools), with a total of 34,922 students. The instrument used was based on the international protocol adopted in the European School Survey Project on Alcohol and Other Drugs (ESPAD) study. The questionnaire’s completion was made online through a dedicated web site, after the delivery of anonymous username and password to each student. In order to reduce the differences in response times between consumers and non-consumers, all the questions in the questionnaire were obligatory. However, each student could interrupt the completion of the questionnaire at any time. The not completed questionnaires were removed from the analysis. The data collected were examined to exclude any unreliable or irrelevant responses: 2700 questionnaires were rejected because they were answered by students outside the age group considered in the survey (15- to 19-year-olds); another 343 questionnaires were rejected because respondents had not completed the sections on gambling or psychotropic substance use; and 218 were omitted because they contained answers that were judged scarcely plausible. This left 31,661 questionnaires considered eligible for the study.

### Variables

For the purposes of the present study, to be defined as ‘gamblers’, respondents had to report having been involved in some form of gambling at least once in the previous year. The SOGS-RA (South Oaks Gambling Screen - Revised for Adolescents) was only given to students who have indicated that they have gambled at least one game in the last 12 months and have been used to examine respondents’ gambling behavior [13]. This validated instrument includes 12 items (scored in the total range from 0 to 12), and identifies three types of gambler, described as: ‘not at risk’ (SOGS-RA score = 0–1); ‘at-risk’ (SOGS-RA score = 2–3); and ‘problem gambling’ (SOGS-RA score ≥ 4). Students who reported having no experience of gambling in the previous year were defined as “never gambled”.

The other variables measured were:

- leisure time activities: “internet surfing”,“playing with the computer”, “playing sports” *(yes/no)*;
- experience in the previous year of: “Getting into a fight”, “Accident or injury”, “Worsening academic achievement”, “Going to the ED (Emergency Department)”, “Having unprotected sex”, “Feeling guilty after sex”, “Pulling stunts” *(yes/no)*;
- school marks, as a dummy *(poor/average/good or very good);*
- substance abuse behavior: “Smoked at least once in the last month” *(yes/no)*, “Drank alcohol at least once in the last month” *(yes/no)*, “Got drunk at least once in the last month” *(yes/no)*, “Smoked cannabis at least once in the last month” *(yes/no)*;
- socioeconomic level: “financial resources*” (more than friends/same as friends/less than friends);* “Given money by parents” *(yes/no)*; “Money spent each week” *(None/€1–50/>€50);*
- social relationships: “Relationship with: mother, father, friends, classmates, teachers” (*satisfactory/neither satisfactory nor unsatisfactory/unsatisfactory);*
- familiar experience of gambling: *“*Mother, father, siblings, grandparents, uncles, or other relatives who gamble” *(yes/no).*
- psychological distress (*high/low),* dichotomized at 25% percentiles of the scores calculated using the SPSD scale (Student Population Survey Distress’s scale) built from nine items belonging to three dominions (Energy, Emotional Stability, Impulsivity and Risk-Taking) from which it is expected the maximal indirect relation to wellbeing state and therefore as complement, to distress; this scale has been validated for use in Italy by Grossi et al. [14]

### Statistical analysis

A bivariate analysis on each of the above-described variables and gambling status was run, distinguishing the sample by gender. A set of Pearson’s chi squared tests was used to highlight any associations between gambling and the other variables.

Furthermore a logistic regression analysis stratified by gender was conducted to assess the association between outcome (gambling status = no or not at risk vs at risk or problem gambling defined as above by SOGS-RA score) and independent predictors. To test the model for multi-collinearity, we calculated the variance inflation factor (VIF), which amounted to 1.62, demonstrating that there was no collinearity among the variables considered.

All *p*-values reported are two-sided and results with p-values below 0.05 were considered statistically significant. Statistical analyses were performed using the SPSS software 18.0.

## Results

**Fig. 1:**
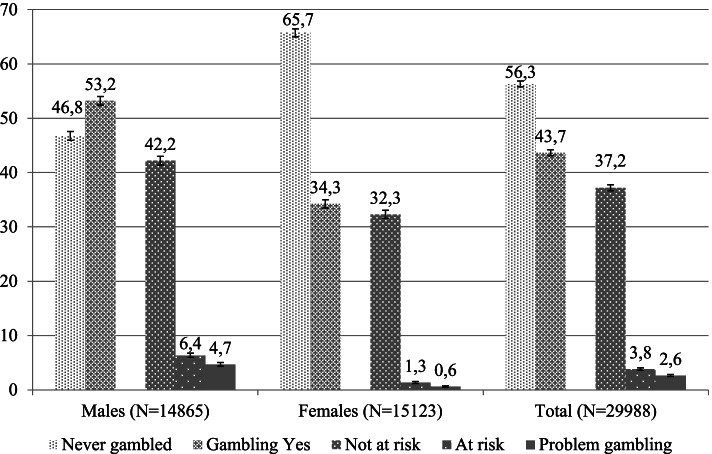
Prevalence of gambling by gender. Percentages (%) and numbers, *p*-value*. * all *p*-value < 0.001

Figure 1 provides details of the gambling behavior of our sample of male and female adolescents: 53.2% of the males, and 34.3% of the females had gambled at least once during the previous year. Males were more likely to be at risk gamblers (males: 6.4%, CI 6.0–6.8; females: 1.3%, CI 1.2–1.5), or problem gamblers (males: 4.7%, CI 4.3–5.0; females: 0.6%, CI 0.5–0.7).

Table 1 shows the results of the bivariate analysis between gambling behavior and the different covariates.

**Table 1:** Bivariate analysis between gambling at risk or problematic and the different covariates. Percentage %, *p*-value

Risk factors		Males ***N*** = 14,865			Females ***N*** = 15,123		
		Gambling at risk or problematic			Gambling at risk or problematic		
		Yes (7915)	No (6950)	***p***-value	Yes (5180)	No (9943)	***p***-value
**Activities and behaviors**							
Leisure activities	Internet surfing	99.1%	97.6%	< 0.001	99.2%	98.2%	< 0.001
	Playing with videogames	95.6%	91.4%	< 0.001	83.4%	74.4%	< 0.001
	Playing sports	94.0%	89.3%	< 0.001	85.1%	82.4%	< 0.001
Things happening at least once in previous year	Getting into a fight	32.8%	23.1%	< 0.001	16.9%	11.9%	< 0.001
	Accident or injury	24.0%	19.1%	< 0.001	18.8%	13.6%	< 0.001
	Worsening academic achievement	45.7%	39.6%	< 0.001	45.8%	41.7%	< 0.001
	Going to ED	18.7%	15.4%	< 0.001	17.7%	14.4%	< 0.001
	Having unprotected sex	18.3%	13.2%	< 0.001	17.0%	12.2%	< 0.001
	Feeling guilty after sex	8.3%	6.9%	0.001	7.3%	5.6%	< 0.001
	Pulling stunts	28.5%	20.5%	< 0.001	16.4%	11.4%	< 0.001
**School marks**							
School marks	Poor	17.5%	14.6%	< 0.001	9.9%	10.1%	0.002
	Average	66.3%	63.3%		64.9%	62.1%	
	Good/very good	16.2%	22.1%		25.2%	27.8%	
**Substance abuse behavior**							
Smoking at least once in previous month		41.3%	32.4%	< 0.001	42.0%	36.8%	< 0.001
Drinking alcohol at least once in previous month		71.5%	58.3%	< 0.001	61.4%	50.7%	< 0.001
Getting drunk at least once in previous month		20.0%	15.8%	< 0.001	16.6%	13.4%	< 0.001
Smoking cannabis at least once in previous month		21.3%	16.6%	< 0.001	13.8%	11.4%	< 0.001
**Socioeconomic level**							
Perceived family income	More than friends	12.0%	13.6%	0.007	10.8%	10.1%	0.104
	Same as friends	79.8%	77.8%		79.7%	81.1%	
	Less than friends	8.2%	8.6%		9.5%	8.8%	
Pocket money from parents		62.7%	56.1%	< 0.001	65.4%	61.7%	< 0.001
Money spent each week	None	7.2%	16.7%	< 0.001	9.7%	16.4%	< 0.001
	€1–50	88.1%	79.2%		87.9%	81.6%	
	> €50	4.7%	4.1%		2.4%	2.1%	
**Social relationships**							
Relationship with mother	Satisfactory	84.5%	82.9%	0.028	78.6%	79.6%	0.289
	Neither satisfactory nor unsatisfactory	9.6%	10.6%		12.9%	12.5%	
	Unsatisfactory	5.9%	6.5%		8.5%	7.9%	
Relationship with father	Satisfactory	78.3%	78.1%	0.767	67.2%	69.2%	0.026
	Neither satisfactory nor unsatisfactory	11.4%	11.8%		16.5%	16.0%	
	Unsatisfactory	10.2%	10.1%		16.3%	14.9%	
Relationship with friends	Satisfactory	89.2%	86.3%	< 0.001	85.1%	85.5%	0.759
	Neither satisfactory nor unsatisfactory	6.9%	8.6%		9.9%	9.5%	
	Unsatisfactory	3.8%	5.2%		5.0%	5.0%	
Relationship with classmates	Satisfactory	78.1%	75.4%	< 0.001	68.8%	69.3%	0.337
	Neither satisfactory nor unsatisfactory	15.8%	16.6%		22.1%	21.2%	
	Unsatisfactory	6.1%	8.0%		9.1%	9.5%	
Relationship with teachers	Satisfactory	46.9%	53.2%	< 0.001	46.9%	52.6%	< 0.001
	Neither satisfactory nor unsatisfactory	36.3%	31.4%		39.8%	35.3%	
	Unsatisfactory	16.8%	15.4%		13.3%	12.1%	
**Familiar experience of gambling**							
Gambling in family	Mother	18.0%	12.4%	< 0.001	27.1%	11.6%	< 0.001
	Father	41.4%	22.3%	< 0.001	42.3%	23.7%	< 0.001
	Siblings	19.6%	8.5%	< 0.001	20.9%	8.4%	< 0.001
	Grandparents	17.2%	14.4%	< 0.001	22.1%	13.8%	< 0.001
	Uncles or other relatives	36.4%	27.2%	< 0.001	39.8%	29.4%	< 0.001
**Psychological distress**							
Low psychological distress		27.0%	31.6%	< 0.001	16.5%	22.0%	< 0.001

Table 2 shows the results of the stratified logistic regression. For both genders, gambling was positively associated with: leisure time spent on videogames, internet surfing, playing sports, getting into a fight, having unprotected sex, pulling stunts, drinking alcohol at least once in the previous month, having not a satisfactory relationship with teachers, receiving pocket money from parents, spending each week much money and having someone in the family (father, sister/brother, other relatives) who gambles. On the other hand, having poor or average school marks, going to ED in the previous year, smoking at least once in the previous month, having dissatisfied with relationships with father and having a lower family income than their friends was only associated with gambling in boys. Having an accident or injury in the previous year and having a mother who gambled was associated only in girls with higher odd of at risk or problem gambling behavior. A low psychological distress is protective only in girls for risk of gambling.

**Table 2:** Logistic regression stratified by gender between outcome variable (gambling = at risk or problem gambling defined as above by SOGS-RA score) and covariates. Odds ratios, 95% confidence intervals, *p*-value

		Males (***N*** = 14,865)				Females (***N*** = 15,123)			
		OR	95% CI		***p***-value	OR	95% CI		***p***-value
			Lower limit	Upper limit			Lower limit	Upper limit	
**Activities and behaviors**									
Internet surfing		**1.58**	**1.17**	**2.15**	**0.003**	**1.87**	**1.29**	**2.69**	**0.001**
Playing with videogames		**1.78**	**1.53**	**2.06**	**< 0.001**	**1.63**	**1.49**	**1.78**	**< 0.001**
Playing sports		**1.58**	**1.39**	**1.80**	**< 0.001**	**1.18**	**1.07**	**1.30**	**0.001**
Getting into a fight in previous year		**1.27**	**1.17**	**1.38**	**< 0.001**	**1.16**	**1.04**	**1.29**	**0.010**
Having accident or injury in previous year		1.02	0.93	1.12	0.655	**1.19**	**1.08**	**1.32**	**0.001**
Going to ED in previous year		**1.12**	**1.01**	**1.23**	**0.027**	1.08	0.98	1.20	0.115
Having unprotected sex in previous year		**1.12**	**1.01**	**1.25**	**0.029**	**1.24**	**1.11**	**1.38**	**< 0.001**
Pulling stunts in previous year		**1.13**	**1.03**	**1.23**	**0.007**	**1.13**	**1.02**	**1.27**	**0.026**
**School marks**									
School marks	Good/very good	1.00				1.00			
	Poor	**1.30**	**1.15**	**1.48**	**< 0.001**	0.88	0.76	1.02	0.080
	Average	**1.23**	**1.12**	**1.35**	**< 0.001**	1.04	0.95	1.13	0.423
**Substance abuse behavior**									
Smoking at least once in previous month		**1.10**	**1.01**	**1.20**	**0.037**	0.94	0.86	1.02	0.144
Drinking alcohol at least once in previous month		**1.39**	**1.28**	**1.50**	**< 0.001**	**1.31**	**1.21**	**1.43**	**< 0.001**
**Socioeconomic level**									
Family income	More than friends	1.00				1.00			
	Same as friends	1.08	0.92	1.26	0.355	0.91	0.77	1.08	0.268
	Lower than friends	**1.17**	**1.05**	**1.30**	**0.004**	0.88	0.78	1.00	0.051
Pocket money from parents		**1.18**	**1.09**	**1.27**	**< 0.001**	**1.11**	**1.03**	**1.20**	**0.007**
Money spent each week	None	1.00				1.00			
	€1–50	**1.96**	**1.75**	**2.20**	**< 0.001**	**1.51**	**1.34**	**1.69**	**< 0.001**
	> €50	**1.80**	**1.47**	**2.21**	**< 0.001**	**1.45**	**1.11**	**1.89**	**0.006**
**Social relationships**									
Relationship with mother	Satisfactory	1.00				1.00			
	Neither satisfactory nor unsatisfactory	0.83	0.73	0.95	0.005	0.94	0.84	1.06	0.319
	Unsatisfactory	0.86	0.73	1.03	0.093	0.94	0.81	1.09	0.402
Relationship with father	Satisfactory	1.00				1.00			
	Neither satisfactory nor unsatisfactory	0.98	0.87	1.11	0.760	0.94	0.84	1.04	0.228
	Unsatisfactory	**1.14**	**1.00**	**1.31**	**0.046**	1.02	0.92	1.14	0.702
Relationship with classmates	Satisfactory	1.00				1.00			
	Neither satisfactory nor unsatisfactory	0.95	0.85	1.05	0.280	1.03	0.94	1.13	0.546
	Unsatisfactory	0.84	0.71	0.99	0.043	0.95	0.82	1.11	0.523
Relationship with teachers	Satisfactory	1.00				1.00			
	Neither satisfactory nor unsatisfactory	**1.16**	**1.07**	**1.26**	**< 0.001**	**1.11**	**1.02**	**1.20**	**0.014**
	Unsatisfactory	1.11	1.00	1.24	0.051	1.12	0.99	1.27	0.073
**Familiar experience of gambling**									
Mother gambles		0.95	0.85	1.06	0.359	**1.89**	**1.72**	**2.08**	**< 0.001**
Father gambles		**2.17**	**2.01**	**2.35**	**< 0.001**	**1.73**	**1.60**	**1.87**	**< 0.001**
Siblings gamble		**2.14**	**1.92**	**2.38**	**< 0.001**	**2.16**	**1.95**	**2.40**	**< 0.001**
Grandparents gamble		0.85	0.77	0.94	0.002	**1.22**	**1.11**	**1.35**	**< 0.001**
Uncle or other relatives gamble		**1.36**	**1.26**	**1.47**	**< 0.001**	**1.38**	**1.28**	**1.49**	**< 0.001**
**Psychological distress**									
Low psychological distress		0.94	0.87	1.01	0.108	**0.84**	**0.76**	**0.92**	**< 0.001**

In bold *p* < 0.05; adjusted also for the following variables (not significant association): worsening academic achievement, feeling guilty after sex, getting drunk at least once in previous month, consuming cannabis at least once in previous month, relationship with friends

## Discussion

The present study showed that male secondary-school students are more frequently gamblers than their female counterparts, and are more likely to be at risk or problem gamblers. In our representative sample of Italian adolescents nationwide, this study also found similarities and differences between male and female adolescent gamblers in terms of their environmental, behavioral, social and psychological risk factors.

Our results are similar to those of a previous study on Italian adolescents and an international study, which found that 55% of male minors and 35% of female minors had gambled at least once [15], and that boys gambled more than girls in a sample of high-school students [16, 17]. The frequency of gambling experiences emerging in our sample is in line with international cross-sectional research indicating that boys gamble more frequently than girls, and are more likely to have gambling-related problems. Judging from the literature, boys are also less likely than girls to consider frequent gambling a risky activity, and more likely to have confidence in their personal gambling skills [10, 18, 19].

In our study, perceived level of financial income was associated with gambling in males. A low perceived income is known to predict more frequent gambling in adults [20] however the study evidenced that money received from parents is associated with gambling in both genders. Similarly, in literature the amount of children’s pocket money has often emerged as a key predictor of gambling and problem gambling. The more money children have, the more likely they are to gamble [21, 22]. Monitoring and containing the amount of money at an adolescent’s disposal for no specific purpose should therefore be considered a valid preventive strategy for parents in both genders.

Little research has been done on the influence of family cohesion on adolescents’ gambling behavior. A strong family environment is known to be a protective factor for adolescents [23]. In our sample, a dissatisfied relationship with father was associated with adolescent gambling in males. Casey and coll. Found that male gamblers had higher levels of conflict in their families than male non-gamblers, whereas female gamblers and non-gamblers did not show such a clear association with their family’s influence [24]. As in our study, Chalmers and Willoughby examined whether the association between parent–adolescent relationships and gambling outcomes differed by gender [25]. They found evidence of the quality of relationships with parents differentiated between low- and high-risk adolescent gamblers, but only for females. They also found evidence of other parental variables being more influential and predicting gambling behavior among female adolescents.

Low anxiety levels have revealed a protective effect on female adolescents in stratified analysis. Consistently one study found anxiety trait associated with adolescent gambling problems, but only among females [26]. One of the goals of both preventive and therapeutic strategies should be to establish the underlying causes of stress and anxiety, and to rebuild healthy interpersonal relationships to remove the detrimental psychological substrate.

Our findings indicated a strong association, for both genders, between father’s, siblings’ and other relatives’ gambling habits and the offspring’s experimenting with gambling. Instead mother’s gambling habit was positively associated only in female. Several authors have underscored the link between young people’s gambling behavior and the gambling habits of their families [27]. For instance, Vachon et al. found youth gambling frequency related both to their parents’ gambling frequency and to the severity of the parents’ gambling problems [28]. Vitaro and Wanner reported similar results, founding that non-problem parental gambling predicted early gambling for boys and girls [29]. Unlike other adolescent risk behaviors, parents often approve and may even be involved in their children’s gambling activities [30, 31]. Parents’ involvement in their children’s gambling reflects parental approval, which has been found associated with higher prevalence of gambling and gambling-related problems among adolescents, giving adolescents the impression that gambling is a socially acceptable and harmless activity [32]. Even if it has not been examined in the empirical literature, siblings’ and extended family members’ attitudes to gambling might influence adolescent gambling.

Internet surfing and playing with videogames were associated with gambling in both genders in our study. Previous research had instead suggested that male frequent videogame players are at greater risk of developing problem gambling habits [33]. The global growth in gambling, coupled with the rising popularity of the internet and various digital technologies, has induced the gambling industry to invest heavily in internet gambling [34]. It has been argued that the internet could easily focus obsessive and/or compulsive behaviors [35]. Research in a number of different national settings has identified that rates of problem gambling amongst young people may be higher among those who gamble on the internet than for those who only gamble offline [36–42].

Finally, in our sample, pulling stunts, getting into a fight, and having unprotected sex were all found associated with gambling in both genders. Previous studies had consistently found an association between adolescent gambling and antisocial or delinquent behavior [43, 44]. However conversely a study on college athletes reported that female (but not male) problem gamblers were more likely to have multiple sexual partners and unprotected sex [45]. Several studies have confirmed the link between impulsiveness and gambling in young children and adolescents. Chambers and Potenza suggested that a common impulsivity trait, rooted in this neurodevelopmental stage, underlies problem gambling and common comorbid psychiatric disorders in adolescents, who exhibit reward sensitivity and deficits in decision-making [46]. In fact, neurodevelopmental models of impulsivity suggest that the immaturity of the brain circuits governing motivation places adolescents at higher risk of experimenting and developing problems with risk-taking behavior [46, 47].

The present study has several limitations, primarily relating to the fact that our data were obtained from a sample of adolescents attending school, which means that those who dropped out of school at 16 years old (non-completing their compulsory education in Italy), who might be at greater risk of gambling problems, were not considered. Our sample is therefore only representative of Italian school goers. A second limitation lies, as with other national prevalence studies, in that the findings are based on self-reports and may consequently underestimate our respondents’ gambling behavior. On the other hand, assuring the respondents’ anonymity and confidentiality, and administering the survey in a controlled environment enhance the likelihood of obtaining accurate informations [48]. Third, the cross-sectional design of the study prevented us from identifying any cause-effect relationships between the variables, though the consistency of our findings with those of other studies on the associations considered should suffice to support the development of a greater public health awareness of the need to prevent any involvement of adolescents in gambling.

## Conclusion

In conclusion, understanding gender-related differences, and how they emerge in younger people at the start of their gambling careers, can provide suggestions on how best to implement prevention strategies for individuals, their families and the wider community. Programs to prevent substance use and abuse should be multifaceted and include efforts to prevent gambling with a gender perspective approach.

## Data Availability

The datasets analysed during the current study are not publicly available but are available from the corresponding author on reasonable request.

## Abbreviations

SPS-DAP: Department for Anti-drug Policies’s Student Population Survey
ESPAD: European School Survey Project on Alcohol and Other Drugs
SOGS-RA: South Oaks Gambling Screen - Revised for Adolescents
ED: Emergency Department
SPSD scale: Student Population Survey Distress’s scale
VIF: Variance inflation factor
CI: Confidence interval
